# Portuguese Validation of the TAPQoL: A Health-Related Quality of Life Instrument for Children Aged 0–6 Years

**DOI:** 10.3390/ejihpe14020027

**Published:** 2024-02-16

**Authors:** Ana Ferraz, Martim Santos, M. Graça Pereira

**Affiliations:** Psychology Research Centre (CIPsi), School of Psychology, University of Minho, 4710-057 Braga, Portugal; anasofiaferraz93@gmail.com (A.F.); martimsantos@email.com (M.S.)

**Keywords:** TAPQoL, validity, preschool children, health-related quality of life, acute lymphoblastic leukemia, burn injuries

## Abstract

In Portugal, there are few generic and specific instruments to assess health-related quality of life (HRQoL) in children, especially those of preschool age. This study aimed to adapt and validate the Portuguese version of the Preschool Children Quality of Life Questionnaire (TAPQoL) in a community and clinical sample of children aged 0–6 years. The parents of 409 healthy children and 137 children undergoing treatment for burns and acute lymphoblastic leukemia completed the TAPQoL and were assessed on psychological morbidity and family functioning. Exploratory and confirmatory factor analyses were performed, as well as analysis of the psychometric properties as shown by internal consistency measures, convergent validity, and average variance extracted. Confirmatory factor analysis confirmed an 11-factor structure with good psychometric properties. The current version of the TAPQoL is a valid and reliable instrument for assessing HRQoL in Portuguese preschool children in community and clinical settings.

## 1. Introduction

Quality of life (QoL) is crucial in medical research [1], particularly as health professionals recognize its importance in assessing both life expectancy and health-related quality of life (HRQoL) [2]. Thus, HRQoL is central in evaluating contemporary medicines and healthcare interventions [3].

Self-report measures of health status have been developed for adults (e.g., SF-36) [4], and both child self-report and parent-proxy report measures are available for the pediatric population [5]; however, a small number of them include younger children [6]. In Portugal, the interest in assessing HRQoL at a pediatric age is still relatively recent [7] and even the international literature remains scarce on this topic [8]. As a result, there are few generic and specific instruments to assess HRQoL in children that are validated for the Portuguese population [9], especially those of preschool age (e.g., FS II-R) [10]. In fact, most measures are developed for children aged 8 years and above [9]. Existing preschool instruments (e.g., FS II-R) lack a multidimensional measure of QoL, and that is the reason why several authors recommend prioritizing a developmentally sensitive, integrated, and multidimensional approach to health outcome measurement in order to accurately capture several aspects of health and illness [11].

However, methodological problems associated with preschool age seem to underlie the absence of age-specific HRQoL measures [12]. The children’s lack of ability/resources to fill in the questionnaires requires their completion by parents, who play a dual role in this process, as legal representatives and respondents. In fact, children under 7 years old are at the preparation cognitive stage, leading to limited understanding due to their inability to perform various logical operations [13].

Lansky et al. [14] pioneered the formal assessment of pediatric HRQoL using measures for parents and physicians. Discrepancies between parents’ reports and children’s/adolescents’ self-reports, especially on subjective measures, are evident in the literature [15]. Additionally, in order to address the challenges of encompassing the entire developmental range (0–18 years old) in a QoL instrument, it is essential to identify specific age ranges based on several health-related domains during instrument development [12].

Nowadays, the decrease in mortality in several chronic diseases requires the evaluation of treatments focused on the state of functional health, HRQoL, and well-being, emphasizing the emerging need for multidimensional instruments [12]. Furthermore, with the increase in young people with chronic diseases and the general scarcity of children’s QoL measures, the need for measures used as indicators of health status has been reinforced [10].

Certain pediatric diseases (e.g., cancer) and physical injuries (e.g., burns) are more prevalent in young children [16], with acute lymphoblastic leukemia (ALL) being the most common type of childhood cancer between two and five years of age [17], and burns being more frequent in children under five years [18]. Despite the reduction in mortality associated with pediatric burn injuries and ALL, there are several long-term consequences, with a significant impact on the HRQoL of these populations [19,20]. Both ALL and burn injuries have similar medical events, including hospitalizations and invasive/painful procedures, being intensive and distressing experiences [16]. Indeed, the literature has shown that a significant percentage of children with burn injuries and ALL continue to show a significant decrease in long-term QoL [21,22] and lower QoL levels when compared to healthy children [23,24]. Therefore, the assessment of pediatric QoL in these clinical conditions is essential to promote health, prevent traumatic responses, and improve healthcare and medical care, over time [25], growing in significance as a secondary treatment outcome [24]. In such young children, proxy measures should be used, being a valuable means of acquiring information about children who are unable to provide reliable self-reports due to their age or cognitive/health limitations [26].

The impact of pediatric burns and ALL diagnosis at an early age may trigger a range of children’s psychosocial problems, including psychological morbidity [27,28] and traumatic symptoms [27,29], negatively influencing their QoL [30,31]. Moreover, there seems to be a relationship between the parents and children’s psychological symptoms, impacting the children’s QoL [32,33]. In this context, family functioning plays a crucial role in children’s QoL, being an important predictor of their emotional functioning [28].

In a recent systematic review focused on QoL assessment instruments at early pediatric age, fifteen generic QoL instruments were identified; however, a significant proportion were aimed at children over five years of age [8]. The same authors emphasize the need for future studies to develop multidimensional measures of HRQoL for children, especially in the age group from zero to three years old, being sensitive to specific developmental aspects that instruments with a wide age range are not able to capture.

One of the instruments to assess the HRQoL of preschool-age children is the TNO-AZL Preschool Children Quality of Life Questionnaire (TAPQoL), based on parental self-report [12]. TAPQoL assesses functional problems weighted by the degree to which the child expresses negative emotions toward such problems. This multidimensional instrument consists of 12 scales (stomach problems, skin problems, lung problems, sleeping, appetite, problem behavior, positive mood, anxiety, liveliness, motor functioning, social functioning, and communication), with higher scores indicating better HRQoL. In the original version, with both preterm children and the general population sample, the unidimensionality of the individual scales was confirmed.

TAPQoL has been translated and validated in several languages such as Chinese [34], Spanish [35], Brazilian [36], Korean [37], and Malay [38]. Most of the versions showed similar properties to the original version, except for the Korean and Malay which found an 11-factor structure. In addition, TAPQoL has shown strong validity and psychometric performance in assessing both infants [39] and preschool children [34], and clinicians have utilized this instrument to evaluate patients with chronic and traumatic health conditions [40,41]. Thus, the acceptance of TAPQoL among clinicians and the general population has been extensive, showing that it is a reliable and valid instrument that may be used in clinical and research settings to assess HRQoL among preschool children [38].

Due to the lack of validated health and morbidity measures in preschool-age children in Portugal, this cross-sectional study aims to translate, adapt, and validate the TAPQoL, in a sample of healthy children and a sample of children undergoing treatment for ALL or unintentional burn injuries, aged 0–6 years old.

## 2. Materials and Methods

### 2.1. Translation Process

The Guidelines for the Process of Cross-Cultural Adaptation of Self-Report Measures by Beaton et al. [42] were used to translate the questionnaire. The English version of the TAPQoL was translated into Portuguese by an expert QoL researcher and a parent. A third researcher assessed the disparities and consolidated them into a unified version. Subsequently, back translation from Portuguese to English was performed by the two independent translators. A comparison of both versions was made by a third researcher, and after a brief discussion regarding the subtle discrepancies, a final version was reached. The final Portuguese version was assessed through an interview with parents regarding its comprehension and cultural adaptation.

### 2.2. Participants and Procedure

The sample included 409 parents of healthy young children (community sample) and 137 parents of children undergoing treatment for unintentional burns and ALL (clinical sample). Data were collected from the community regarding the healthy sample and from four Portuguese central hospitals regarding the clinical sample, between March2021 and October 2023. The inclusion criteria were as follows: (i) parents of healthy children, with ALL or unintentional burns; (ii) parents being legal guardians and primary caregivers of the child; (iii) children aged 0–6 years. Exclusion criteria included not speaking Portuguese. Regarding the healthy sample, participants were recruited from the community, i.e., nursery and kindergarten educational establishments through email and social networks. Those who met the inclusion criteria and agreed to participate in the study answered the questionnaires through an online survey software: Qualtrics XM^TM^ (March 2021 to October 2023). Regarding the clinical sample, participants were recruited and answered the questionnaires during the inpatient or outpatient phases. The research protocol included the study goals, data confidentiality, voluntary participation, and the informed consent form. This study was approved by the Ethics Committee for Research in Social and Human Sciences of a major public university and by the ethics committees of the four included hospitals. The permission to translate and validate the instruments was granted by the original authors.

### 2.3. Instruments

#### 2.3.1. Sociodemographic Questionnaire

This questionnaire assesses sociodemographic variables in parents and children, such as sex, age, marital status, education, and children’s state (being healthy or undergoing treatment).

#### 2.3.2. TNO-AZL Preschool Children Quality of Life Questionnaire (TAPQoL) [12]

The TAPQoL is a multidimensional instrument with 43 items that measure parents’ perceptions of HRQoL in preschool children. The instrument consists of 12 scales. For five of the scales (problem behavior, positive mood, anxiety, liveliness, and social functioning), items include one question reporting a specific complaint or limitation, scored on a 3-point Likert-type scale (never, occasionally, and often). For the other seven scales (stomach problems, skin problems, lung problems, sleeping, appetite, motor functioning, and communication), items consist of two questions: the first one assesses the presence of a complaint or limitation, scored on a 3-point Likert-type scale (never, occasionally, and often); the second one assesses the well-being of the child related to such problem or limitation, measured on a 4-point Likert-type scale (fine, not so good, quite bad, and bad). Three scales (social functioning, motor functioning, and communication) are only relevant for children aged 18 months and older. Scale scores are calculated by adding up the item scores within each scale and subsequently converting the raw scale scores into a linear 0–100 scale, as well as the total score, with higher scores indicating better QoL. In the original version, Cronbach’s alpha ranged from 0.43 to 0.84 for the general population.

#### 2.3.3. Hospital Anxiety and Depression Scale (HADS) [43,44]

This instrument that evaluates psychological morbidity includes 14 items distributed across two subscales: anxiety and depression. Answers are given on a 4-point Likert-type scale ranging from 0 to 3. High scores indicate greater psychological morbidity. In the original version, Cronbach’s alpha was 0.76 for the anxiety subscale and 0.81 for the depression subscale. In this study, only the total scale was used with a Cronbach’s alpha and McDonald’s omega of 0.89.

#### 2.3.4. Family Assessment Device–General Functioning Subscale (FAD-GF) [45,46]

In this study, the global scale of the FAD was used to assess the perception of the global functioning of the family through 12 items. Answers are given on a 4-point Likert-type scale, from 1 (strongly agree) to 4 (strongly disagree). High scores indicate troubled family functioning. In the original version, Cronbach’s alpha was 0.92. In this study, both the Cronbach’s alpha and McDonald’s omega were 0.88.

### 2.4. Data Analysis

Statistical analyses were performed using SPSS and SPSS AMOS (software 29.0 version). To describe the sociodemographic characteristics of parents and children, descriptive analysis was performed. Internal consistency of the TAPQoL was examined using Cronbach’s alpha, McDonald’s omega, and composite reliability (CR), with coefficients ≥ 0.7 suggesting good reliability [47]. Composite reliability was calculated through Raykov’s formula [48].

Exploratory factor analysis (EFA) and confirmatory factor analysis (CFA) were performed to obtain the final factor structure of the Portuguese version of the TAPQoL. The following fit indices were considered: the ratio of chi-square over the number of degrees of freedom (χ^2^/DF) (values less than 5.0 are associated with good ft); the root mean square error of approximation (RMSEA, values below 0.08 are acceptable); Tucker–Lewis Index (TLI); and the comparative fit index (CFI) (values ≥ 0.95 reflect a better fit) [47].

Convergent validity evidence was assessed using the coefficients of the average variance extracted (AVE) above 0.5 [49] and Pearson correlations between the TAPQoL total score and scales, and HADS and FAD-GF scores.

## 3. Results

### 3.1. Participants

The sample included parents (546) among which 89% were mothers, with 86.3% being married/cohabited, 57.3% attended higher education, and 78.6% were employed. The mean age was 34.27 (SD = 5.62). Of the total sample, 74.9% of the children belonged to the community healthy group, and the remaining to the clinical group. The majority were boys (55.5%) and the children’s mean age was 2.89 (SD = 1.66). The sample included 48.7% of children with siblings, and most families (73.8%) lived in urban areas. Table 1 describes the sociodemographic characteristics of parents and children.

### 3.2. Internal Consistency

The Cronbach’s alpha ranged from 0.62 to 0.95 and the McDonald’s omega ranged from 0.65 to 0.95 for the instrument scales, with values of 0.83 for the total scale. For most scales, those values were above 0.70, except for the scales measuring stomach problems, skin, and anxiety (above 0.60). Additionally, CR ranged between 0.61 and 0.92 for the instrument scales, with 0.98 for the total scale (Table 2).

### 3.3. Principal Characteristics of the Structural Model

To assess the adequacy of the sample, principal component analysis (PCA) was performed. The results of Bartlett’s sphericity test (χ^2^ = 11,100.286, *p* < 0.001) and the Kaiser–Meyer–Olkin sample adequacy test confirmed the sample adequacy to proceed with factor analysis (0.87). According to the eigenvalues of Kaiser’s criterion (above 1.0 is a good indicator of latent factors), a solution of 11 factors was found, since the two scales of “Positive mood” and “Liveliness” in the original version were joined as one single scale in the Portuguese version, called “Positive mood and Liveliness”. This 11-factor structure explained 71.88% of the total variance. Eight items showed factor loading higher than 0.30 on more than one scale, although the highest loading was on their original scales. Similarly, item 29 showed factor loading higher than 0.30 on two scales, but the authors chose to keep it in the original scale (Table 3). Also, the unidimensionality of each scale was confirmed by PCA. The 11-factor structure was confirmed by CFA, indicating acceptable goodness-of-fit indices. The fit indices were χ^2^/DF = 2.83, CFI = 0.88, TLI = 0.86, RMSEA = 0.058, and 90% CI [0.055, 0.061].

### 3.4. Correlation between Items and Subscales

The correlation coefficients between each item and the corresponding scale were all above 0.70 (*p* < 0.001), except for item 9 of the stomach scale, items 18, 19, and 23 of the problem behavior scale, and item 40 of the communication scale. However, all of these correlation coefficients were above 0.60.

The correlation coefficients between the 11 scales of the TAPQoL ranged on average between 0.01 and 0.64. Most inter-scale correlation coefficients were found to be below 0.5, except for the positive mood and liveliness scale and the motor functioning (r = 0.59) and social functioning (r = 0.64) scales (Table 4).

### 3.5. Convergent Validity

Indices of convergent validity indicated no validity concerns, with AVE being greater than 0.50, except for the anxiety and behavior scale (Table 4). TAPQoL total score correlated negatively with HADS as well as its scales. TAPQoL total score and its scales also correlated negatively with FAD-GF, except for the stomach and motor functioning scales. Only the total scale score and the scale measuring behavior problems showed correlations greater than the recommended threshold of 0.30 with HADS (Table 5).

## 4. Discussion

In Portugal, there are no validated instruments to assess HRQoL in young children. Thus, the aim of the present study was the validation of the TAPQoL in a Portuguese sample of children aged from 0 to 6 years old. To accomplish this goal, EFA and CFA were conducted to determine the final factor structure of the Portuguese version of the TAPQoL.

The Portuguese version retained most of the items from the original version [12]; however, the items from the positive mood and liveliness scales were merged into one scale, resulting in an 11-factor structure with reasonable goodness-of-fit indices. Regarding the CFI and TLI values, despite being lower than 0.95 in this study, they may be considered acceptable since, according to Portela [50], only values lower than 0.80 reflect a bad fit. Also, the unidimensionality of each scale was confirmed. This structure was also found in the Korean [37] and Malay [38] versions, and another study that assessed the validity of the Spanish version of the TAPQoL in a sample of Colombian preschool children [51] found the same emerging scales. According to the authors, this finding could be attributed to cultural differences in the perception of liveliness and positive emotions, which were interpreted as similar. It seems that Portuguese parents consider liveliness (“energetic”, “active”, and “lively”) and positive mood (“in good spirit”, “cheerful”, and happy”) as being the same without differentiation.

The internal consistency of the total scale was good (0.83) and comparable to the Malay version [38]. Regarding the 11 subscales, most of them showed Cronbach’s alpha above 0.70, but three of them revealed low values, especially the stomach scale, skin scale, and anxiety scale. However, these findings are not unique to the present study [36,37], suggesting that these scales are somehow problematic in other languages as well, rather than specific to the Portuguese translation. In fact, according to Fekkes et al. [12], the results may be related to the low prevalence and variance of those problems in the sample.

The low correlation coefficients between the 11 scales suggest that the TAPQoL effectively measures several aspects of children’s HRQoL, with the scales being distinct and non-overlapping compared to the original version [12] and other adapted versions [34].

In terms of convergent validity, the results indicated good validity in the Portuguese version. Also, the TAPQoL had significant correlations with HADS and FAD-GF, except for the stomach and motor functioning scales. Parents’ psychological morbidity and poorer family functioning were significantly associated with worse child HRQoL. It is well documented in the literature that parents are a critical factor in promoting their children’s adaptive outcomes [52], and previous studies corroborate these findings, emphasizing that healthy family functioning appears to be a key contributor to a child’s better HRQoL [53,54,55].

Also, the way in which a family handles stressful situations significantly influences the well-being of all its members [56]. Indeed, the diagnosis of a chronic disease poses several adjustments and challenges, impacting parental well-being [57] and enhancing the risk for psychological distress [58]. Parental symptoms of anxiety and depression have been negatively related to children’s HRQoL across a wide range of health conditions in this age group [33,59]. Moreover, other studies have found that parents’ psychological functioning was linked to the child’s emotional, cognitive, and behavioral responses and, consequently, impacts the child’s overall functioning [60]. Furthermore, since the child’s HRQoL was assessed by parents, it is essential to understand their emotions to mitigate the risk of bias in the parents’ reports [38]. Thus, monitoring parents’ psychological symptoms and understanding the factors that precipitate these symptoms may have a protective and lasting impact on children’s health outcomes, especially given the existing evidence of a potential relationship between the parents’ psychological functioning and the children’s overall well-being [52,60].

Additionally, family functioning was not associated with the stomach and motor functioning scales, suggesting that the perception of the global functioning of the family and children’s stomach and intestinal problems as well as gross motor problems may be independent constructs. In addition, this study used the general functioning scale of FAD, which is more focused on global family functioning (healthier versus poorer functioning), rather than specific dimensions.

### Limitations

This study presents some limitations that need to be acknowledged, such as the sample size and proxy reports. Although proxy reports are the only way to assess HRQoL in young children, some concerns arise. In fact, the perceptions of children may not be accurately reflected in proxy responses given by their parents, as several factors such as mental health and life experiences may influence parents’ responses [38]. Nevertheless, primary caregivers continue to be regarded as reliable sources of information [34]. Moreover, the data were collected during the COVID-19 pandemic, so the results should be interpreted with caution. In fact, a recent systematic review confirmed that the evidence for significant differences in children’s HRQoL before and after the pandemic was not robust [61]. Thus, further evidence is needed, especially from longitudinal studies, to clarify causal relationships. In addition, this study used a convenience sample. The sample size of both groups should also be considered, and therefore, the results should be interpreted cautiously. Also, the present study included more mothers (486; 89%) than fathers. Future studies should include more fathers to obtain a more balanced proxy reports from both parents regarding their children’s perceived HRQoL.

Additionally, the measurement invariance between groups (healthy sample vs. clinical sample as well as between children under 18 months vs. children 18 months and above) was not assessed, since the number of participants in each group did not exceed the recommended minimum size of *N* = 200 [62]. Future studies should assess invariance in the factor model across groups and comparisons between groups.

As proposed by Rajmil et al. [35], a shorter version of the TAPQoL that uses summary scores to provide a concise overview of the child’s HRQoL would be highly beneficial.

## 5. Conclusions

The Portuguese version of the TAPQoL is a valid and reliable tool for assessing HRQoL in infants, toddlers, and preschoolers with healthy and clinical conditions. Similar to the Korean and Malay versions, in the Portuguese version, positive mood, and liveliness scales emerged as a single scale, resulting in an 11-factor structure. Despite that, this version remains representative of the original version, maintaining the general consistency and multidimensionality of the instrument. Moreover, the Portuguese version of the TAPQoL, in general, showed greater internal consistency than the original version.

According to the results, it is important to consider parental and family variables when assessing the HRQoL of young children. This validation study constitutes a “window of opportunity” to provide pediatric health professionals/researchers with a useful tool for early screening and monitoring of children’s developmental/behavioral problems in order to inform clinical practice focused on promoting children’s HRQoL.

Finally, this is the first validation of an instrument to measure the HRQoL of Portuguese children aged 0 to 6, in community and clinical settings.

## Figures and Tables

**Table 1:** Sample characteristics of parents and children.

	Parents (Respondents)				Children			
	N (%)	Mean (SD)	Min	Max	N (%)	Mean (SD)	Min	Max
Sample								
Healthy sample					409 (74.9)			
Clinical sample					137 (25.1)			
Burn injury					100 (18.3)			
ALL					37 (6.8)			
Sex								
Female	486 (89.0)				243 (44.5)			
Male	60 (11.0)				303 (55.5)			
Age		34.27 (5.62)	21	62		2.89 (1.66)	0.08	6.00
Female		34.05 (5.35)	21	56		3.22 (1.65)	0.17	6.00
Male		36.00 (7.27)	27	62		2.63 (1.62)	0.08	6.00
Marital Status								
Single	57 (10.4)							
Married/Cohabited	471 (86.3)							
Divorced	16 (2.9)							
Widower	2 (0.4)							
Education								
With higher education	313 (57.3)							
Without higher education	233 (42.7)							
Professional Status								
Employed	429 (78.6)							
Unemployed	113 (20.7)							
Retired	4 (0.7)							
Living area								
Urban	403 (73.8)							
Rural	143 (26.2)							
Siblings								
Yes					266 (48.7)			
No					280 (51.3)			

Note: ALL = acute lymphoblastic leukemia.

**Table 2:** Internal consistency of the TAPQoL.

Scales	Number of Items	Cronbach’s α	McDonald’s ω	CR
Total	43	0.83	0.83	0.98
Stomach	3	0.62	0.65	0.75
Skin	3	0.68	0.69	0.83
Lungs	3	0.82	0.83	0.90
Sleeping	4	0.79	0.79	0.81
Appetite	3	0.82	0.83	0.86
Motor Functioning	4	0.95	0.95	0.90
Positive mood and Liveliness	6	0.93	0.93	0.92
Anxiety	3	0.67	0.68	0.61
Problem behavior	7	0.84	0.84	0.86
Social Functioning	3	0.92	0.92	0.82
Communication	4	0.84	0.86	0.87

Note: CR = composite reliability.

**Table 3:** Factor analysis of the TAPQoL.

Scales and Items	1	2	3	4	5	6	7	8	9	10	11
Factor 1. Stomach											
Item 1	**0.80**	−0.02	0.13	0.10	0.02	0.18	−0.14	0.04	0.12	−0.03	−0.04
Item 2	**0.64**	0.06	0.07	0.22	0.08	0.25	−0.17	0.18	0.01	−0.21	0.06
Item 9	**0.68**	−0.02	−0.01	−0.01	0.25	−0.02	−0.05	0.13	0.13	0.07	0.08
Factor 2. Skin											
Item 3	−0.03	**0.81**	0.04	0.07	0.10	−0.11	0.05	−0.01	0.07	−0.03	0.09
Item 4	−0.01	**0.78**	−0.03	0.04	−0.06	0.02	−0.07	−0.09	0.17	−0.07	0.01
Item 5	0.06	**0.77**	0.11	−0.02	0.01	0.03	−0.09	0.09	0.12	0.12	−0.07
Factor 3. Lungs											
Item 6	0.03	0.05	**0.84**	0.07	0.02	−0.04	−0.04	0.08	0.00	0.00	0.07
Item 7	0.04	0.05	**0.88**	0.07	0.06	0.16	0.03	0.00	0.07	0.01	0.00
Item 8	0.09	0.01	**0.88**	0.03	0.10	0.08	−0.02	0.02	−0.02	−0.07	0.03
Factor 4. Sleeping											
Item 10	0.29	0.05	0.16	**0.70**	0.00	0.02	−0.04	0.01	0.18	−0.03	0.06
Item 11	−0.02	0.05	0.13	**0.62**	0.18	−0.02	−0.18	0.23	0.11	0.06	0.14
Item 12	−0.05	0.04	−0.03	**0.75**	0.13	0.11	0.00	0.05	0.21	−0.06	0.14
Item 13	0.09	−0.02	−0.01	**0.80**	0.08	0.09	−0.03	0.00	0.15	−0.05	0.08
Factor 5. Appetite											
Item 14	0.17	−0.01	0.05	0.06	**0.84**	0.09	−0.07	0.11	0.09	0.00	0.14
Item 15	0.19	0.04	0.03	0.14	**0.82**	0.05	−0.15	0.08	0.02	−0.02	0.15
Item 16	−0.04	0.03	0.12	0.16	**0.79**	0.19	0.02	−0.06	0.15	−0.08	0.05
Factor 6. Motor functioning											
Item 36	0.12	−0.04	0.09	0.05	0.11	**0.79**	−0.42	0.03	0.06	−0.10	0.11
Item 37	0.14	0.02	0.11	0.08	0.14	**0.84**	−0.35	0.05	0.04	−0.04	0.08
Item 38	0.03	−0.05	0.02	0.08	0.10	**0.85**	−0.26	0.10	0.06	−0.09	0.17
Item 39	0.10	−0.02	0.04	0.05	0.07	**0.84**	−0.18	0.12	0.07	−0.16	0.15
Factor 7. Positive mood and liveliness											
Item 24	−0.06	−0.03	0.03	−0.10	−0.06	−0.05	**0.86**	0.03	−0.17	0.10	0.02
Item 25	−0.06	−0.02	0.04	−0.08	−0.15	−0.11	**0.85**	−0.05	−0.14	0.16	0.02
Item 26	−0.10	−0.06	−0.01	−0.03	−0.01	−0.18	**0.87**	−0.05	−0.08	0.18	0.01
Item 30	−0.07	−0.04	−0.10	0.03	0.06	−0.42	**0.70**	−0.16	0.02	0.14	−0.04
Item 31	−0.07	−0.02	−0.05	0.01	−0.01	−0.44	**0.74**	−0.14	−0.04	0.17	−0.03
Item 32	−0.09	−0.01	−0.02	−0.05	−0.05	−0.25	**0.85**	−0.03	−0.02	0.22	−0.08
Factor 8. Anxiety											
Item 27	0.09	−0.08	0.10	0.15	0.16	0.29	0.03	**0.64**	0.17	−0.09	0.07
Item 28	0.21	−0.02	−0.02	0.13	0.06	0.17	−0.26	**0.46**	0.48	−0.17	0.11
Item 29	0.31	0.08	0.05	0.10	−0.04	0.04	−0.32	**0.64**	0.18	−0.08	−0.03
Factor 9. Problem behavior											
Item 17	0.05	0.04	0.02	0.11	0.14	0.04	−0.10	0.04	**0.70**	0.06	0.09
Item 18	−0.09	0.14	0.02	−0.07	0.06	−0.01	−0.21	0.21	**0.69**	0.01	0.04
Item 19	0.19	0.15	0.04	0.10	−0.01	0.19	−0.12	0.01	**0.65**	−0.08	0.15
Item 20	0.09	0.12	0.04	0.04	0.03	0.06	−0.11	0.18	**0.73**	−0.10	0.09
Item 21	0.22	0.03	0.02	0.18	−0.02	0.15	−0.11	−0.12	**0.70**	−0.06	0.02
Item 22	0.01	0.03	−0.01	0.14	0.05	−0.13	0.11	−0.11	**0.71**	0.04	0.01
Item 23	−0.08	0.01	−0.03	0.16	0.02	−0.02	0.07	0.15	**0.64**	−0.06	0.01
Factor 10. Social functioning											
Item 33	−0.02	0.03	0.03	0.01	−0.02	−0.23	0.49	−0.06	−0.06	**0.71**	−0.06
Item 34	−0.04	0.01	−0.05	0.01	−0.03	−0.10	0.41	−0.08	−0.06	**0.83**	−0.10
Item 35	−0.07	0.01	−0.05	−0.13	−0.09	−0.15	0.38	−0.09	−0.09	**0.79**	−0.13
Factor 11. Communication											
Item 40	−0.17	−0.11	0.04	0.09	0.06	0.18	0.01	0.17	0.15	0.06	**0.61**
Item 41	0.01	0.05	0.07	0.05	0.08	0.10	0.00	−0.02	0.05	−0.18	**0.83**
Item 42	0.12	0.02	0.01	0.10	0.11	0.04	−0.03	−0.03	0.09	−0.06	**0.88**
Item 43	0.10	0.06	0.00	0.14	0.09	0.11	−0.05	−0.01	0.08	−0.03	**0.84**

Note: Bold indicates item saturation.

**Table 4:** Pearson correlation coefficients between the 11 scales of the TAPQoL.

Scales	1	2	3	4	5	6	7	8	9	10	11
1. Stomach	-										
2. Skin	0.05	-									
3. Lungs	0.16 ***	0.12 **	-								
4. Sleeping	0.28 ***	0.13 **	0.18 ***	-							
5. Appetite	0.22 ***	0.12 **	0.23 ***	0.28 ***	-						
6. Motor functioning	0.37 ***	−0.01	0.17 ***	0.23 ***	0.30 ***	-					
7. Positive mood and liveliness	0.23 ***	0.04	0.03	0.08	0.11 *	0.59 ***	-				
8. Anxiety	0.40 ***	0.07	0.14 ***	0.21 ***	0.26 ***	0.43 ***	0.33 ***	-			
9. Problem behavior	0.20 ***	0.23 ***	0.12 **	0.31 ***	0.26 ***	0.18 ***	0.14 ***	0.50 ***	-		
10. Social functioning	0.26 ***	0.01	0.07	0.16 ***	0.16 ***	0.44 ***	0.64 ***	0.37 ***	0.18 ***	-	
11. Communication	0.15 **	0.05	0.10 *	0.29 ***	0.28 ***	0.29 ***	0.11 *	0.21 ***	0.22 ***	0.22 ***	-

Note: * *p* < 0.05; ** *p* < 0.01; *** *p* < 0.001.

**Table 5:** Convergent validity indicators.

Scales	AVE	HADS	FAD-GF
Total	0.59	−0.40 **	−0.29 ***
Stomach	0.50	−0.20 ***	−0.08
Skin	0.62	−0.15 ***	−0.13 **
Lungs	0.75	−0.12 **	−0.16 ***
Sleeping	0.52	−0.25 ***	−0.15 **
Appetite	0.67	−0.22 ***	−0.18 ***
Motor functioning	0.69	−0.19 ***	−0.07
Positive mood and liveliness	0.66	−0.24 ***	−0.24 ***
Anxiety	0.34	−0.27 ***	−0.18 ***
Problem behavior	0.48	−0.35 ***	−0.25 ***
Social functioning	0.61	−0.23 ***	−0.22 ***
Communication	0.63	−0.18 ***	−0.12 *

Note: AVE = average variance extracted. * *p* < 0.05; ** *p* < 0.01; *** *p* < 0.001.

## Data Availability

As part of the ethical approval process and due to the sensitive nature of the questions asked in this study, survey respondents were assured that raw data would remain confidential and would not be shared or transferred to any third party.

## List of Abbreviations

**Abbreviation**	**Definition**
ALL	Acute Lymphoblastic Leukemia
AVE	Average Variance Extracted
CFA	Confirmatory Factor Analysis
CR	Composite Reliability
EFA	Exploratory Factor Analysis
FAD-GF	Family Assessment Device–General Functioning Subscale
FS II-R	Functional Status II-R
HADS	Hospital Anxiety and Depression Scale
HRQoL	Health-Related Quality of Life
PC	Principal Component Analysis
QoL	Quality of Life
RMSEA	Root Mean Square Error of Approximation
SF-36	36-Item Short Form Health Survey
TAPQoL	TNO-AZL Preschool Children Quality of Life Questionnaire
TLI	Tucker–Lewis Index
